# Neurobiological Synergy of Plant and Animal Sources of Omega‐3 and Exercise in Aging: Implications for Molecular Signaling, Memory, Spatial Learning, and Brain Function

**DOI:** 10.1002/fsn3.70866

**Published:** 2025-09-04

**Authors:** Yikuang Hao, Sima‐sadat Sabihi

**Affiliations:** ^1^ Sports Institute Henan University of Science and Technology Luoyang Henan China; ^2^ Food Security Research Center Isfahan University of Medical Sciences Isfahan Iran

**Keywords:** aging, exercise, fish oil, flaxseed oil, neurodegenerative diseases

## Abstract

Aging is associated with cognitive decline, impaired spatial learning, and diminished brain function, significantly impacting quality of life (QoL). Emerging evidence suggests that lifestyle interventions, like omega‐3 fatty acids (FAs) intake and regular exercise, can mitigate these age‐related deficits by targeting key molecular pathways implicated in oxidative damage, inflammation, and reduced fibrinolytic activity. By doing so, omega‐3 FAs, principally eicosapentaenoic acid and docosahexaenoic acid, influence signaling pathways that enhance synaptic plasticity, prevent apoptosis, and promote neurogenesis. Similarly, moderate exercise stimulates neurogenesis, synaptic plasticity, and mitochondrial biogenesis. The synergistic effects of omega‐3 and exercise may further amplify neuroprotective mechanisms by enhancing hippocampal function, improving cerebral blood flow, and modulating gut‐brain axis interactions. This review explores the molecular crosstalk between exercise and omega‐3 FAs, highlighting their joint impact on spatial learning, memory retention, and brain health in aging. Additionally, we discuss potential translational implications for neurodegenerative disease prevention and cognitive longevity. An understanding of these molecular mechanisms could pave the way for individualized interventions to optimize brain function, thus improving quality of life in aging subjects.

## Graphical abstract

Omega‐3 fatty acids from animal sources, combined with exercise, modulate key molecular pathways to enhance neurogenesis, synaptic plasticity, and antioxidant defenses. These interventions also increase tissue plasminogen activator activity, promoting both amyloid‐β degradation and proBDNF cleavage into BDNF, thereby reducing neurodegeneration and improving cognitive function. Together, they offer synergistic neuroprotective effects that may mitigate aging‐related brain decline.

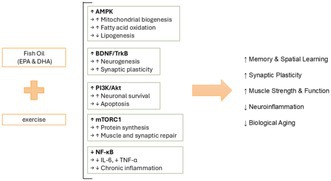

## 1 Introduction

Age‐related alterations in the human brain occur across functional, structural, and metabolic dimensions. These changes include reductions in gray matter volume and white matter integrity (Gunning‐Dixon et al. 2009; Kennedy and Raz 2009), and neurotransmitter abnormalities, such as serotonin, dopamine, and receptor binding and acetylcholine synthesis (Schliebs and Arendt 2011; Wu et al. 2005), which are often accompanied by cognitive decline, particularly in executive functions. Also, executive functions represent higher‐level cognitive processes responsible for regulating and coordinating lower‐level cognitive abilities and purposeful behaviors (Banich et al. 2009; Lopez‐Franco et al. 2018), including navigating complex environments. Specific constituents of executive functions, like working memory (Holtzer et al. 2006, 2007), suppression control (Hausdorff et al. 2005), and divided attention (Sheridan et al. 2003), play critical roles in gait regulation. Impaired executive functions have been related to gait abnormalities and an augmented risk of falls (Scherder et al. 2011).

Building upon prior research, physical exercise, including active video games, exerts valuable impacts on the well‐being of elderly subjects (Stanmore et al. 2017; Eggenberger et al. 2016; Schättin et al. 2016). Similarly, omega‐3 fatty acids (FAs) have been exhibited to help cognitive roles in older subjects (Phillips 2017). Omega‐3 FAs, commonly found in flaxseed and fish oils, have been proposed as potential nutritional supports that may enhance the positive impacts of physical activity. These FAs are crucial for brain energy metabolism, maintaining the function and structure of neuronal plasma membranes, and promoting cerebral blood flow (Janssen and Kiliaan 2014; Dawczynski et al. 2013). Older adults, in particular, may benefit from administration of long‐chain polyunsaturated FAs, as aging is related to a deterioration in their cerebral concentration (Janssen and Kiliaan 2014). Long‐chain polyunsaturated FAs supplementation has been shown to reduce neuroinflammation and restore fibrinolytic activity, thus improving cognitive performance and decreasing vascular risk factors in this demographic (Janssen and Kiliaan 2014; Hoirisch‐Clapauch and Nardi 2015). Among omega‐3 FAs, docosahexaenoic acid (DHA), a crucial constituent of fish oil, is the most abundant in the human brain and plays a strong, irreplaceable role in maintaining integrity and neuronal membrane function, thereby contributing significantly to cognitive health (Phillips 2017; Hertzog et al. 2008). Eicosapentaenoic acid (EPA), another key omega‐3 FA in fish oil, also offers distinct benefits, particularly through its anti‐inflammatory properties. Animal studies further highlight these distinctions: both DHA and EPA stimulate neurite outgrowth during growth phases, but only DHA FAs have demonstrated neuroprotective effects in aged rat brain tissue (Dyall 2015). Importantly, research recommends that the most pronounced cognitive assistance of DHA supplementation may be detected in older individuals without existing cognitive impairments (Dyall 2015). Given that omega‐3 fatty acids, particularly DHA and EPA, have been shown to improve endothelial function and fibrinolytic capacity, and that exercise independently upregulates tPA expression, it is plausible that the combined intervention of omega‐3 intake and physical activity may support Aβ clearance through modulation of the tPA‐plasmin pathway. This interaction represents a promising, underexplored mechanism by which lifestyle factors could mitigate Alzheimer's pathology and warrants further investigation (Melchor et al. 2003).

The rationale for this review stems from the growing need to explore effective strategies to mitigate cognitive decline and promote brain health in aging populations. The world's population is aging with increasing speed; neurodegenerative diseases like Parkinson's, Alzheimer's, and other forms of cognitive impairment are becoming increasingly prevalent. These conditions are associated with significant economic, social, and personal burdens, emphasizing the urgent requirement for therapeutic and preventive interventions. Omega‐3 FAs from plant‐ and animal‐based sources have been shown to have anti‐inflammatory and antioxidant properties, as well as pro‐fibrinolytic activity, all of which improve brain functions by enhancing neurogenesis and neuroplasticity. However, while individual studies have observed the impact of omega‐3 intake on brain health, there is limited understanding of the effects of both animal and plant sources of omega‐3 FAs, especially in the context of aging. Additionally, the function of physical exercise in improving brain function and its potential synergy with omega‐3 supplementation remains underexplored.

Brain‐derived neurotrophic factor (BDNF) and its precursor, pro‐BDNF, are both members of the neurotrophin family and exhibit distinct, often opposing functions. Although pro‐BDNF promotes neuronal apoptosis and long‐term depression (LTD), mature BDNF facilitates neurogenesis, synaptic plasticity, and LTP, both of which are central to memory formation and cognitive flexibility (Mizui et al. 2015; Ikegaya et al. 2002). Emerging evidence suggests that omega‐3 fatty acids not only reduce inflammation and triglyceride levels but also upregulate tissue plasminogen activator (tPA), a protease responsible for cleaving pro‐BDNF into mature BDNF. Omega‐3, by upregulating tPA, increases BDNF levels, which promotes neuroplasticity, enhancing cognition in aging (Kokoli et al. 2017).

Omega‐3 PUFAs and their metabolites not only exert anti‐inflammatory and cardioprotective effects but also enhance tPA activity, thereby contributing to improved fibrinolytic capacity and neurovascular health (Golanski et al. 2021). Importantly, tPA promotes the proteolytic cleavage of proBDNF into mature BDNF (Pang et al. 2004). This distinction is critical because proBDNF is associated with apoptotic signaling and LTD, whereas mature BDNF facilitates neurogenesis, synaptic plasticity, and long‐term potentiation, all of which are essential for cognitive resilience and memory consolidation (Golanski et al. 2021).

The implication is that omega‐3 supplementation may support brain plasticity not only via direct effects on membrane fluidity and signaling but also through an indirect mechanism: boosting tPA activity, which shifts the BDNF equilibrium toward its neuroprotective form. This synergy is further amplified by exercise, which independently increases both BDNF and tPA expression (Pang et al. 2004).

Together, these findings support the hypothesis that omega‐3 fatty acids combined with physical activity may potentiate BDNF‐mediated neuroplasticity through enhanced tPA‐dependent proBDNF cleavage, offering a mechanistic explanation for their cognitive and antiaging benefits (Golanski et al. 2021).

This review aims at critically investigating the neurobiological apparatuses underlying the synergistic effects of exercise and omega‐3 FAs in aging. By focusing on molecular signaling pathways such as BDNF, AMP‐activated protein kinase (AMPK) activation, the mammalian target of rapamycin (mTOR), and synaptic plasticity, this review will provide a comprehensive understanding of how these interventions interact to influence memory, spatial learning, and other brain functions. Furthermore, it will explore the implications of these interventions for aging‐related diseases and propose personalized approaches to optimize cognitive health in elderly populations. Ultimately, the present review seeks to advance the interpretation of how combined dietary and exercise interventions could be used to prevent cognitive decline, thus contributing to healthier aging.

## 2 Memory, Spatial Learning, and Physical Exercise

It is widely accepted that the brain undergoes constant remodeling throughout the entire lifespan (Leuner and Gould 2010; Bruel‐Jungerman et al. 2007). Neurogenesis and neuroplasticity, such as activity‐dependent synaptic changes and restructuring of neuronal networks, enable the central nervous system to acquire novel skills, recall and form memories, reorganize neural circuits in response to external stimuli, and recover from injury (Knaepen et al. 2010; Coelho et al. 2013). The hippocampus, a highly plastic structure, plays a critical role in declarative memory and spatial navigation. It consists primarily of the dentate gyrus and the cornus ammonis (CA2, CA1, CA3) (Alkadhi 2019). Each subregion displays distinct neuroplastic properties and contains unique cell populations that respond differently to physical exercise, thereby contributing in diverse ways to memory and learning (Voss et al. 2013). Studies in mice have shown that voluntary physical activity improves pattern separation abilities, likely caused by exercise‐induced neurogenesis. In humans, Cassilhas et al. (2007) found enhancements in both long‐ and short‐term spatial memory in elderly individuals after 6‐month resistance training. Similarly, Erickson et al. (2009) demonstrated that aerobic exercise led to improved temporary spatial memory in association with increased aerobic fitness. The findings were connected to an increased hippocampal volume, which corresponded with the observed cognitive improvements.

## 3 Flaxseed and Its Bioactive Constituents

**FIGURE 1:**
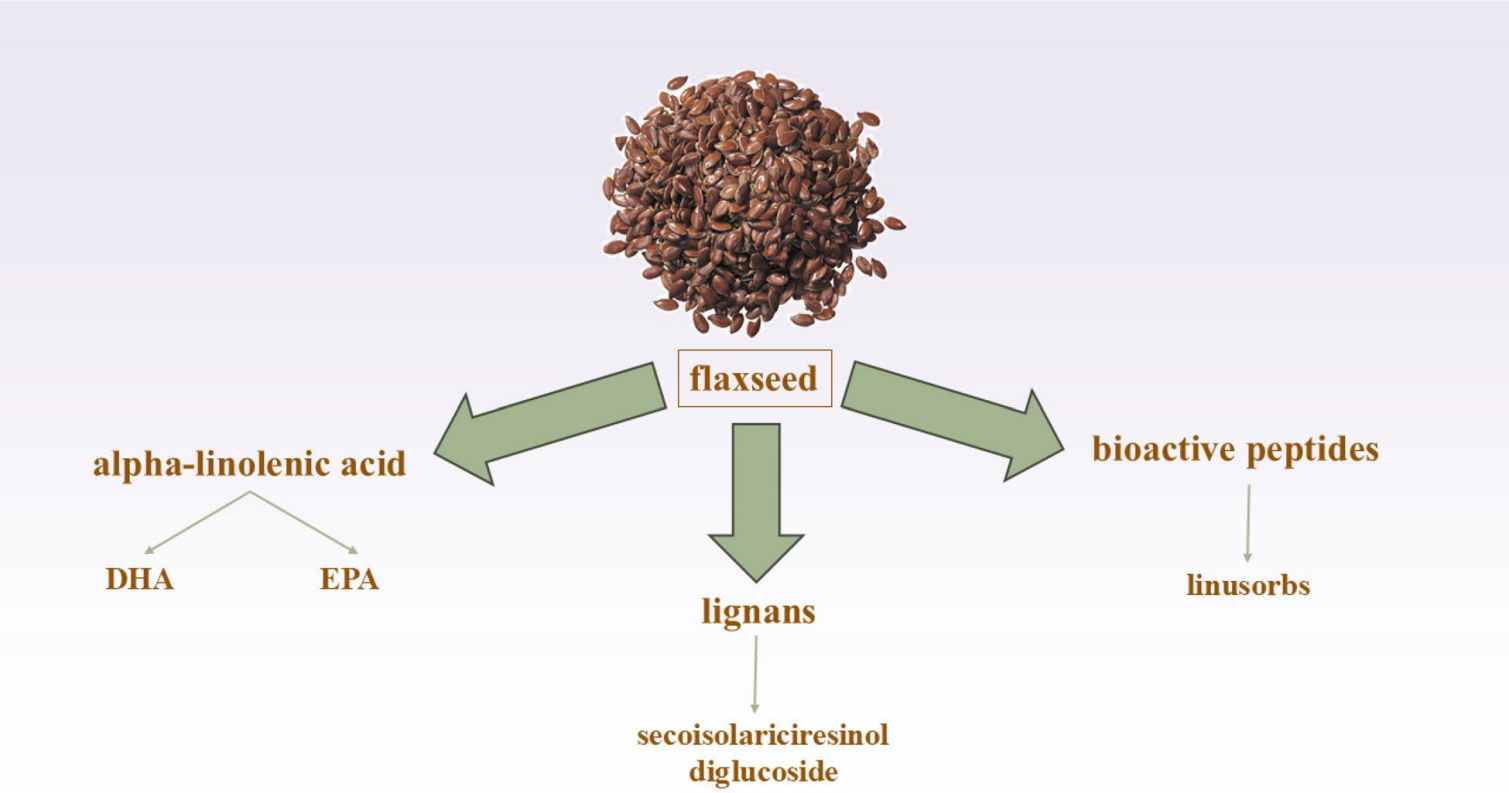
Whole or ground flaxseed are rich in various bioactive compounds that help health‐promotion. Key constituents comprise lignans, alpha‐linolenic acid (ALA), proteins and peptides, dietary fiber, and cyanogenic glycosides. Flaxseed is the richest plant‐based source of ALA, an essential FA that cannot be synthesized in the human body. Flaxseed has high ALA content, as a precursor to omega‐3 FAs, such as DHA and EPA, ALA. Additionally, flaxseeds have lignans, especially secoisolariciresinol diglucoside (SDG).

Whole or ground flaxseed is rich in various bioactive compounds that help health promotion. Key constituents comprise lignans, alpha‐linolenic acid (ALA), proteins and peptides, dietary fiber, and cyanogenic glycosides (Figure 1) (Mueed, Shibli, et al. 2022). ALA is an omega‐3 polyunsaturated fatty acid (PUFA) which constitutes about 22% of whole flaxseed and 50%–60% of flaxseed oil (Hall 3rd et al. 2006; Giada 2010). Flaxseed is the richest plant‐based source of ALA, an essential FA that cannot be synthesized in the human body. Flaxseed has a high ALA content; as a precursor to omega‐3 FAs, such as DHA and EPA, ALA has anti‐inflammatory activity, playing an important role in cardiovascular and neurological health (Goyens et al. 2006; Hussein et al. 2005). Additionally, flaxseeds have lignans, especially secoisolariciresinol diglucoside (SDG). Flaxseeds contain around 75% insoluble fiber and 25% soluble fiber. The soluble fiber promotes intestinal health, helps lower cholesterol concentrations, and aids in regulating blood glucose levels (Singh et al. 2011). Upon digestion, SDG is changed into secoisolariciresinol, which is further metabolized into enterolactone and enterodiol by gut bacteria (Peterson et al. 2010). SDG is the principal lignan found in flaxseed, representing one of its most biologically significant components (Adolphe et al. 2010). Upon ingestion, SDG undergoes microbial metabolism in the gut to form two key mammalian lignans: enterolactone and enterodiol. These metabolites have been extensively studied for their antioxidant, anti‐inflammatory, and estrogenic properties, which contribute to multiple health benefits, particularly in aging populations. Both enterolactone and enterodiol act as free radical scavengers, helping to neutralize reactive oxygen species (ROS) (Adolphe et al. 2010). This function is crucial in aging, as oxidative stress is a major driver of neurodegeneration and cellular aging. In summary, the transformation of SDG into enterolactone and enterodiol enhances flaxseed's value as a dietary intervention for healthy aging. Their multifaceted biological actions, spanning antioxidant, anti‐inflammatory, and hormone‐modulating effects, make them important mediators in preserving brain function and overall health during aging (Adolphe et al. 2010).

Flaxseed bioactive peptides like linusorbs and proteins demonstrate antioxidant and antihypertensive effects. Although whole flaxseeds provide fiber and lignans, they need to be ground to release ALA (Mueed, Madjirebaye, et al. 2022). Whole flaxseed has a longer lifetime than ground flaxseed, but most of it is excreted undigested. Flaxseed oil is high in ALA but lacks the lignans and fiber found in ground or whole seeds (Yang, Wen, et al. 2021). Since it is sensitive to light and heat, refrigeration is suggested to preserve its potency. Partly defatted flaxseed meal, a by‐product of oil extraction, retains important amounts of fiber, protein, and lignans, making it an important nutritional choice.

## 4 Fatty Acids and Exercise Beneficial Effects in the Brain

Lifestyle interventions prevent age‐related diseases and help maintain a high quality of life (QoL) throughout one's lifetime. Flaxseed has gained attention for promoting longevity, primarily due to its cardiometabolic benefits, exemplified by reduced blood pressure, enhanced lipid profiles, and reduced systemic inflammation. The beneficial effect of flaxseeds on longevity includes a reduced risk of type 2 diabetes mellitus (Bhardwaj et al. 2015; Fekete et al. 2024; Yabluchanskiy et al. 2018). Aging is often accompanied by a persistent, low‐grade inflammatory condition known as “inflammaging,” which acts as a key function in the onset of age‐related cardiometabolic diseases. Flaxseed's ALA plays a role as a precursor to anti‐inflammatory eicosanoids that regulate inflammation, reducing biomarkers like C‐reactive protein (CRP) (Shibabaw 2021). Oxidative damage, resulting from an imbalance between antioxidant defenses and ROS, accelerates cellular aging and damages key cellular components like DNA, proteins, and lipids (Ungvari et al. 2018; Csiszar et al. 2008). ALA and lignans neutralize ROS, safeguarding cellular structures from oxidative damage. By decreasing oxidative damage, flaxseed decreases malondialdehyde (MDA) concentrations, a biomarker of lipid peroxidation. Flaxseed also elevates insulin sensitivity and regulates blood glucose concentrations, with lignans playing a role in improving insulin sensitivity by influencing glucose homeostasis and alleviating hyperglycemia‐induced cellular stress. One of the key mechanisms underlying this effect involves flaxseed's modulation of the gut microbiota: its high soluble fiber and lignan content promote a shift from pro‐inflammatory to anti‐inflammatory microbial populations, such as increased abundance of Lactobacillus and Bifidobacterium species. This microbiota shift reduces systemic inflammation and improves metabolic regulation, thereby enhancing insulin sensitivity (Osmakov et al. 2022; Jang et al. 2022; Kumar and Devaraja 2022). As individuals age, significant changes occur in the gut microbiome, frequently resulting in dysbiosis, which is related to metabolic disorders and inflammation. Flaxseed's fiber content supports a healthy gut microbiome, with soluble fiber being a prebiotic that stimulates the growth of noninflammatory gut bacteria like Lactobacilli and bifidobacteria (Kleigrewe et al. 2022). In addition, lignans and ALA enhance autophagy, a process by which cells eliminate damaged constituents (Hamazaki and Murata 2024).

## 5 The Effects of Flaxseed Oil and Exercise on Brain Function in Aging

**FIGURE 2:**
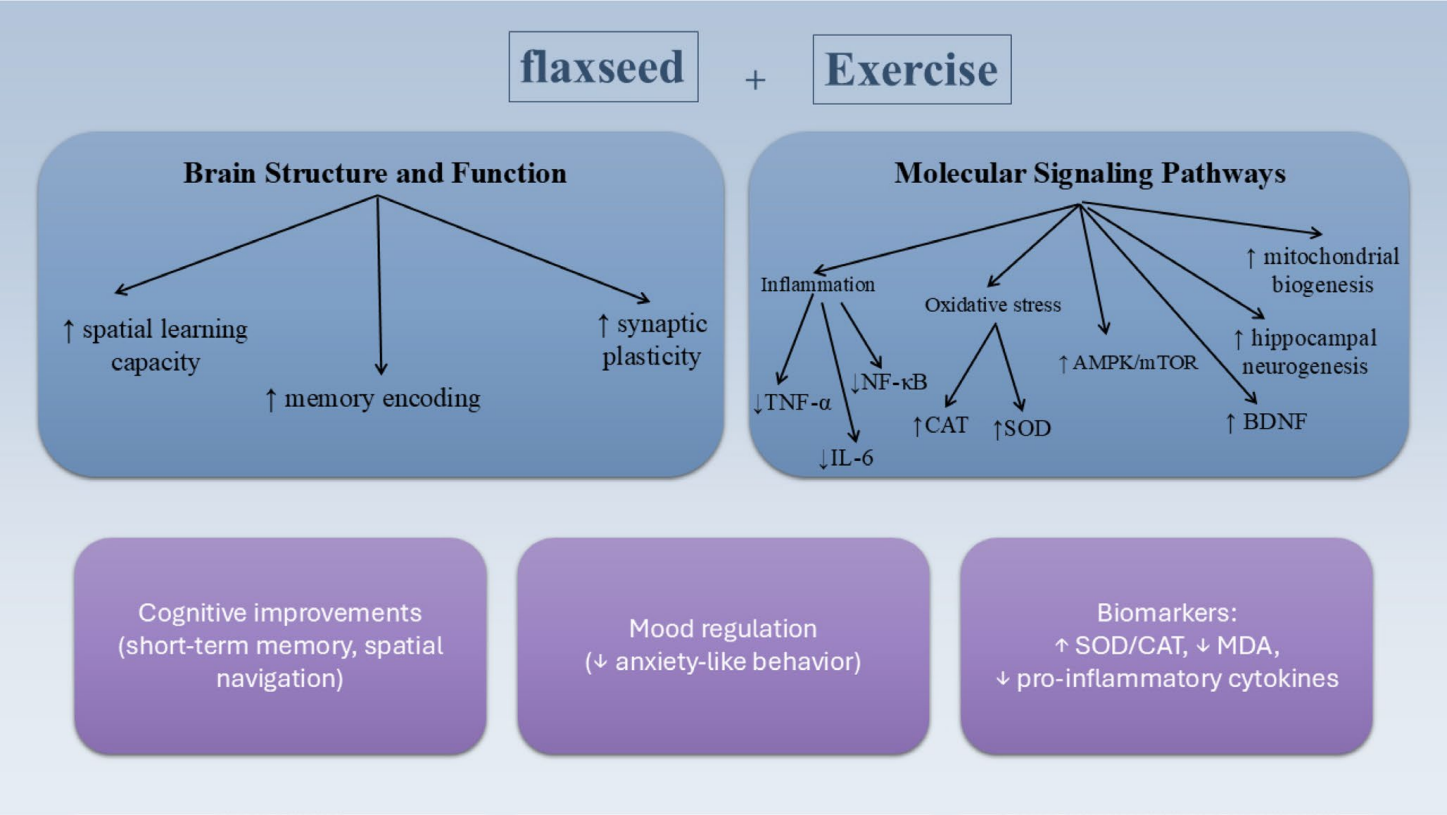
The beneficial effects of flaxseed oil combined with exercise on molecular signaling, memory, spatial learning, and brain function in aging.

Aging as well as neurodegenerative diseases like Parkinson's disease (PD) and Alzheimer's disease (AD) are related to progressive impairments in spatial learning, memory, and other brain functions. These conditions are driven by a complex interplay of oxidative damage, neuroinflammation, and impaired neurotrophic signaling pathways, principally in brain areas like the prefrontal cortex and hippocampus (Mattson 2004; Sebastian Monasor et al. 2020). Mitochondrial dysfunction leads to elevated oxidative stress and decreased energy production, thereby contributing to aging (Figure 2) (Rahman 2020; Jaca et al. 2020; Farooqui and Farooqui 2012).

### 5.1 Exercise and Brain Function Enhancement in Aged Patients

**FIGURE 3:**
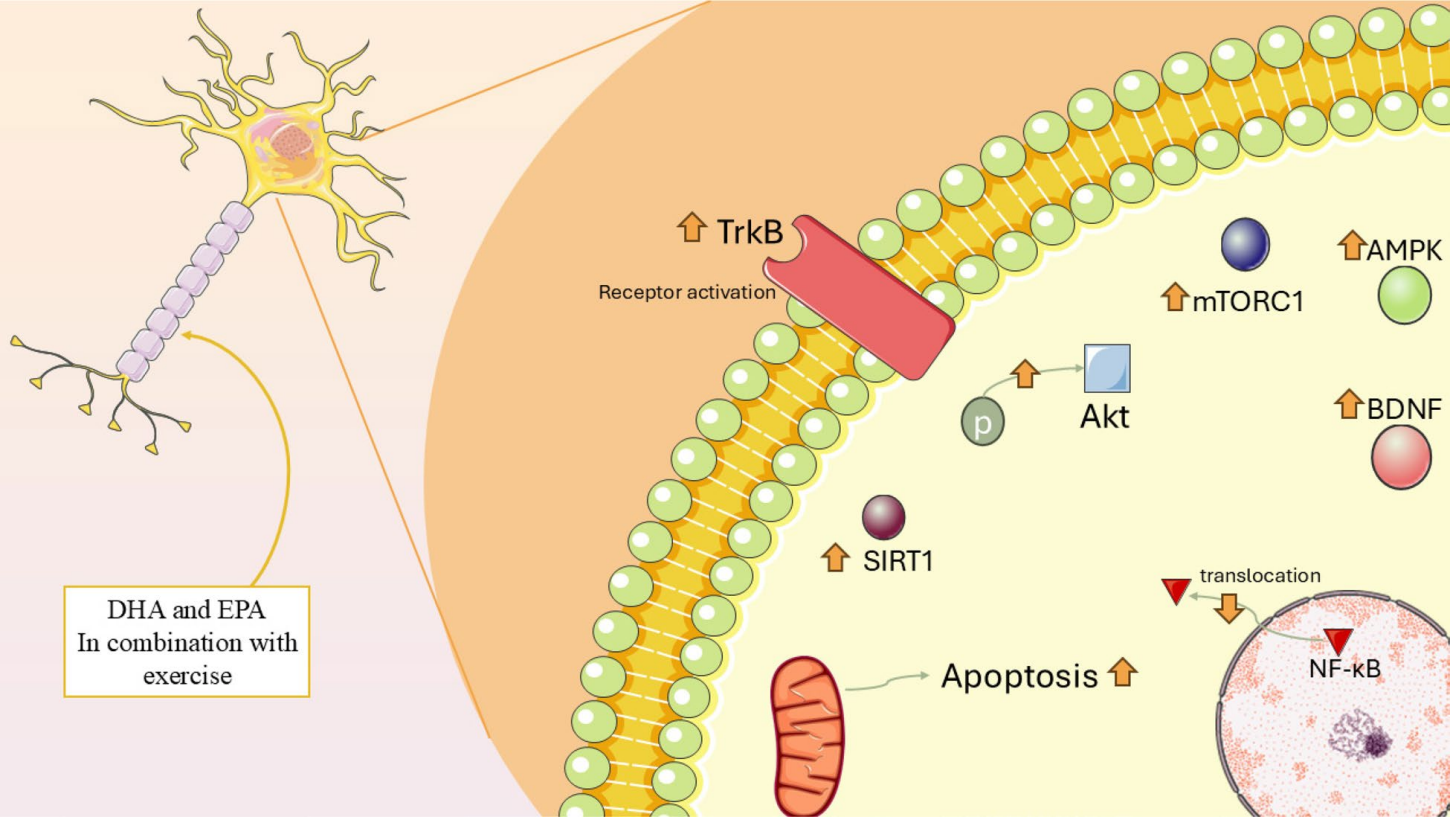
Synergistic effects of animal‐derived omega‐3 fatty acids and exercise on brain health in aging.

Furthermore, moderate physical exercise is well‐known for enhancing BDNF expression, promoting hippocampal neurogenesis and norepinephrine release, and improving cognitive performance across the lifespan (Figure 3) (Cotman et al. 2007; Pelegrino et al. 2025; Venezia et al. 2017). Despite the growing interest in nutritional neuroscience and exercise physiology, few reports assessed the influence of ALA combined with physical exercise on cognitive function related to aging. Firuzya et al. (Jaca et al. 2020) assessed the impact of an 8‐week exercise (resistance training program) combined with flaxseed oil intake on antioxidant markers, specifically superoxide dismutase (SOD) and catalase (CAT), in women aged 50–65 years diagnosed with T2DM. Subjects in the intervention groups engaged in resistance exercises at 40% of their one‐repetition maximum (1‐RM), three times/week for 8 weeks. Those in the supplementation group consumed two capsules of flaxseed oil daily, each containing 200 mg. The findings revealed a significant elevation in CAT levels in the exercise, the flaxseed oil, and the combined intervention groups following the 8‐week program, with no notable changes observed in the control group. Similarly, SOD levels significantly increased in all three intervention groups, whereas the control group showed no such improvement. Notably, the flaxseed oil supplementation group exhibited the greatest increases in both CAT and SOD levels (Firuzyar et al. 2023). In turn, Jahanshir et al. (Farooqui and Farooqui 2012) investigated the influences of combined training and/or flaxseed supplementation on blood pressure and insulin resistance in postmenopausal women. A total of 27 women aged 50–60 years were recruited through convenience sampling. The intervention included aerobic training at 60%–80% of maximum heart rate and resistance training at 60%–80% of one‐repetition maximum. Participants in the supplementation group received 25 g of ground flaxseed daily. The findings demonstrated that the combination of exercise and flaxseed intake led to an improvement in maximal oxygen uptake (VO_2_ max), lowered blood pressure levels, and a reduction in body mass index (BMI) (Jahanshiri and Bijeh 2020) (Table 1).

**TABLE 1:** The impacts of plant and animal sources of omega‐3 combined with exercise on molecular signaling and brain function in aging.

Participants	Dosage and type	Duration	Results	References
Women with type 2 diabetes mellitus	400 mg/day flaxseed capsule	8 weeks	Increased SOD and CAT	Firuzyar et al. (2023)
Postmenopausal women	25 g of the milled flaxseed	8 weeks	Increase maximal oxygen consumption, and decrease in BMI and blood pressures	Jahanshiri and Bijeh (2020)
Older adults	1 g of omega‐3 (330 mg of EPA plus 660 mg of DHA)	3 years	No effect on blood pressures, nonvertebral fractures, physical performance, infection rates, cognitive function	Bischoff‐Ferrari et al. (2020)
Community‐dwelling older females	3 g of fish oil	12 weeks	Increased resting metabolic rate, energy expenditure during exercise, the rate of fat oxidation during rest. Lowered triglyceride levels and increased lean mass and functional capacity	Logan and Spriet (2015)
Older women	2000 mg of omega‐3	8 weeks	Decreased HOMA‐IR index, body fat percentage, body mass index, IL‐6, and TNF‐α	Sobhani et al. (2024)
Older women	3000 mg of omega‐3	12 weeks	Increased isometric knee‐extensor strength, muscle anabolism (mTORC1) and plasma C‐reactive protein remained unchanged by omega‐3, whereas IL‐6 tended to decrease in omega‐3	Dalle et al. (2021)
Community‐dwelling old adults	2.2 g/day of omega‐3	8 weeks	Increased muscle power and reduced inflammation in old men, but not in old women	Haß et al. (2022)
Older adults	13.5 mL of fish oil/day	26 weeks	No effect on neuronal system levels in the brain and behavioral measurements	Schättin et al. (2019)
Older men	3 g/day of omega‐3	12 weeks	No effect on inflammatory markers	Cornish et al. (2018)
Older adults	1 g of omega‐3 (330 mg of EPA plus 660 mg of DHA)	3 years	A small protective effect on slowing biological aging and DNA methylation	Bischoff‐Ferrari et al. (2025)
Aged male Wistar rats	Low dose: 160 mg/kg of body weight High‐dose: 320 mg/kg of body weight		Improved plasma antioxidant capacity and GPx activity, and reduced CAT activity in erythrocytese	Paduchová et al. (2024)
Aged rats	160 and 320 mg/kg		Low dose: reduced locomotor activity. High‐dose reduced anxiety‐like behavior and improved recognition memory	Gajdošová et al. (2024)
Elderly women	2000 mg/day of omega‐3	8 weeks	Reduced ICAM‐1 and VCAM‐1 levels	Arabzadeh et al. (2024)
Elderly women		16 weeks	No effect on BDNF	Daďová et al. (2022)
Older adults	1491 mg DHA+351 mg eicosapentaenoic acid/day	12 months	No effect on cognition and the positive effect on APOE ɛ4 carriers, depression and anxiety scores	Mengelberg et al. (2022)
Aged rats	Soft‐shelled turtle oil		Improved memory and physical strength, and SOD levels	Yang, Yeh, et al. (2021)
Aged obese mice		18 months	Delay NAFLD progression during aging	Yang, Sáinz, et al. (2021)
Old man	2700 mg of omega‐3	2.2 years	Attenuated resistance exercise‐induced apoptosis	Sheikholeslami‐Vatani et al. (2019)

### 5.2 The Antiaging Effects of Fish Oil

An increasing number of individuals are incorporating fish oil supplements, particularly those comprising the omega‐3 FAs of DHA and EPA, into their diets (Calder 2001; Calder and Grimble 2002). These FAs, originally derived from marine algae, accumulate in fish. It was documented that fish oil influences many cardiovascular risk factors (Sidhu 2003). Notably, it lowers triglyceride levels, an effect often combined with an increase in both HDL‐ and LDL‐cholesterol levels (Kris‐Etherton et al. 2003; Harris 1997). Both DHA and EPA contribute to these triglyceride‐lowering effects. In subjects with mild hypertension, but not in healthy individuals, omega‐3 intake has also been linked with reductions in both diastolic and systolic blood pressure (Cabo et al. 2012). Fish oil has cardioprotective effects and may reduce the risk of diabetes, stroke, and AD (Yavari et al. 2025; Coelho‐Júnior et al. 2024). In murine models of atherosclerosis, omega‐3 supplementation has led to reductions in plaque formation. Additionally, it was reported that dietary omega‐3s helped mitigate toxin‐induced neuronal degeneration in an animal model of PD (Bousquet et al. 2008).

## 6 The Favorable Properties of Omega‐3 FAs From Animal Sources Combined With Exercise on Molecular Signaling, Memory, Spatial Learning, and Other Brain Functions

A study by Bischoff‐Ferrari et al. (2020) involving 2157 seniors aged 70 and older found that vitamin D, strength training, and omega‐3 fatty acids had no significant effects on health outcomes such as fractures, blood pressure, infections, cognitive function, or physical performance after three years. Logan and Spriet assessed the impact of fish oil supplementation on (1) substrate oxidation and metabolic rate during rest and after exercise; (2) blood pressure and heart rates during resting and after exercise; (3) body composition; (4) physical function and strength; and (5) blood measures of glucose, triglycerides, insulin, and CRP in older females. A total of 24 females were randomized to 3 g/day of DHA and EPA for 12 weeks or to a control group. The authors concluded that fish oil supplements considerably raised the rate of fat oxidation at rest by 19%, the resting metabolic rate by 14%, and the rate of energy expenditure during exercise by 10%. Consuming fish oil also improved lean mass by 4%, functional capability by 7%, and triglyceride levels by 29% (Logan and Spriet 2015).

An Irani study showed that 2 g omega‐3 FAs combined with exercise has a significant effect on metabolic parameters, reducing body fat percentage, HOMA‐IR, IL‐6, BMI, and TNF‐α (Sobhani et al. 2024). Following exercise, the training + omega‐3 and omega‐3 training groups had lower concentrations of TNF‐α than the placebo group. Moreover, IL‐6 concentrations in the training group were lower than in the placebo group post exercise. Combined training could reduce both IL‐6 and TNF‐α compared to placebo. Unlike omega‐3 alone or combined with training, combined training could decrease TNF‐α levels but not IL‐6 compared to placebo (Sobhani et al. 2024).

A trial studied the impact of omega‐3 intake on muscle inflammation and metabolic responses (anabolic/catabolic processes) after resistance exercise in older adults. Twenty‐three people were assigned to 3 g omega‐3 FAs daily or to a placebo group while undergoing a 12‐week resistance exercise (RE) regimen (three sessions/week). The results indicated that isometric knee‐extensor strength meaningfully was enhanced in the omega‐3 group but not in the placebo group, although both groups showed improvements in leg press strength. Resistance training alone, rather than omega‐3 administration, led to reductions in inflammatory (p65NF‐κB) and catabolic (LC3b and FOXO1) markers, as well as enhancements in muscle quality. However, neither RE nor omega‐3 affected overall muscle volume. Additionally, anabolic signaling pathways (mTORC1) and CRP levels remained unaffected in both groups, although IL‐6 levels tended to decline with omega‐3 intake. These findings suggest that while omega‐3 supplementation may enhance isometric strength gains induced by resistance exercise, it does not further improve the anti‐inflammatory or anti‐catabolic benefits of resistance training. Improvements in strength, a key indicator of sarcopenia, may be attributed more to enhancements in muscle quality rather than increases in muscle mass (Dalle et al. 2021). A German group investigated the impact of an 8‐week intervention combining vibration and home‐based resistance training along with omega‐3 FAs and whey protein on inflammation, muscle power, and muscle‐related markers in community‐dwelling older subjects. The findings demonstrated an increase in IGF‐1 levels with the addition of omega‐3 supplementation. Notably, sex‐specific analysis revealed significant improvements in muscle power, IL‐6/IL‐10 ratio, IL‐6, and HMGB‐1 concentrations exclusively in males who received high‐protein and omega‐3‐enriched dietary intervention (Haß et al. 2022).

A case–control study including 58 Swiss subjects older than 65 years evaluated the impact of exergame combined with fish oil over 26 weeks on behavior and mental health. The authors found no effect (Schättin et al. 2019). A randomized trial in 23 men ≥ 65 years evaluated daily omega‐3 supplementation (3 g/day EPA + DHA) plus progressive resistance training versus training with placebo. Both groups followed the same 12‐week full‐body regimen, with assessments of muscle strength, inflammatory cytokines, body composition, and physical function at baseline and follow‐up. Omega‐3 plus training led to improvements in percent body fat, bone mineral density, lean mass, strength, and functional performance (timed‐up‐and‐go, 6‐min walk). No significant between‐group differences were observed in inflammatory cytokines (Cornish et al. 2018).

A study presented findings, investigating the effects of daily supplementation with omega‐3 FAs (1 g), vitamin D (2,000 IU), and/or participation in a home‐based exercise program on four advanced DNA methylation (DNAm) biomarkers of biological aging, namely GrimAge2 (An advanced DNA methylation clock that estimates biological age and predicts disease risk and mortality using epigenetic markers linked to blood proteins and smoking history), PhenoAge (A DNA methylation measure that reflects physiological health and predicts lifespan by integrating clinical biomarker profiles), GrimAge, and DunedinPACE (A DNA methylation–based metric that estimates the current *rate* of biological aging rather than total biological age), over three years. The analysis revealed that omega‐3 supplementation alone significantly decelerated the GrimAge2, aging‐related DNAm clocks PhenoAge, and DunedinPACE. Additionally, all 3 interventions demonstrated cumulative benefits in reducing PhenoAge (Bischoff‐Ferrari et al. 2025). A preclinical study in Wistar rats examined the effects of omega‐3 fatty acids combined with moderate‐intensity graded aerobic exercise on oxidative stress and antioxidant defenses. Rats were assigned to a sedentary control group, an exercise‐only group, or exercise groups receiving low (160 mg/kg) or high (320 mg/kg) omega‐3 doses. The combined interventions enhanced antioxidant capacity and glutathione peroxidase activity while reducing catalase activity. Findings suggest a synergistic benefit of omega‐3 supplementation with exercise for oxidative stress reduction (Paduchová et al. 2024).

Another study assessed the impact of low or high‐dose omega‐3 FAs intake combined with exercise on cognitive performance and anxiety‐like behaviors of adult Wistar rats. The authors found that exercise combined with high‐dose omega‐3 enhanced recognition memory and reduced anxiety‐like behaviors (Gajdošová et al. 2024). The combination of 2 g omega‐3 daily and physical activity was shown to downregulate biomarkers of cardiovascular disease in elderly women. In that study, there was a substantial reduction in VCAM‐1 and ICAM‐1 after eight weeks, which was accompanied by a significant decrease in body fat percentage and insulin resistance. These findings suggest additive vascular and metabolic benefits from combining exercise with omega‐3 intake (Arabzadeh et al. 2024). A placebo‐controlled Czech study, including 55 elderly women, investigated the impact of an exercise program along with or without omega‐3 FAs intake on short‐term episodic memory. After 16 weeks of functional circuit training twice weekly and Nordic walking sessions once a week, it was found that short‐term memory improved in both groups independent of BDNF levels, with omega supplementation not conferring any additional benefit (Daďová et al. 2022).

A clinical trial was conducted to assess the cognitive impacts of DHA‐rich fish oil intake in older subjects with mild cognitive impairment, and to explore whether the apolipoprotein E (APOE) ɛ4 allele modulates outcomes related to cognition and psychological well‐being. The study involved 72 participants aged 60–90 years from New Zealand, who received either a daily DHA supplement (351 mg EPA and 1491 mg DHA) or a placebo over a 12‐month period. The analysis revealed no significant treatment effects on cognitive performance. However, a decrease in systolic blood pressure was observed in the supplement group, and a significant interaction was found between DHA supplementation and APOE ɛ4 carrier status, indicating improvements in depression and anxiety scores among carriers (Mengelberg et al. 2022). A different study found that in rats with aging induced by D‐galactose, feeding them soft‐shelled turtle oil (SSTO), either alone or combined with swimming exercise, led to improvements in memory and physical strength. The FAs profiles of SSTO observed that unsaturated FAs made up over 70% of the oil, with more than 20% being omega‐3 FAs. Moreover, the combination of SSTO feeding and swimming significantly increased SOD concentrations and helped maintain better blood pressure in the aging rats. Additionally, serum glycogen and dehydroepiandrosterone sulfate levels in the soleus muscle were strongly associated with both SSTO feeding and swimming training (Yang, Yeh, et al. 2021).

A study examined whether DHA intake and aerobic exercise over 18 months, either alone or in combination, could help improve nonalcoholic fatty liver disease (NAFLD) in aging obese mice. The DHA‐enriched diet reduced liver steatosis by decreasing the expression of lipogenic genes and increasing the expression of lipolytic genes, the same occurring with diet‐induced obese mice undergoing exercise. DHA combined with exercise further boosted the expression of fatty acid oxidation regulators. Both exercise alone and combined with DHA significantly reduced the expression of pro‐inflammatory genes in diet‐induced obese mice (Yang, Sáinz, et al. 2021). An Irani group compared the impact of acute resistance exercise only or combined with either branched‐chain amino acids or omega‐3 on markers of apoptosis and cell survival. After one week, exercise only increased cytochrome c, sFasL, and Bax concentrations, but no differences in Bcl‐2 and nuclear factor‐kappa B were found in both arms undergoing supplementation. The study recommended that BCAA or omega‐3 FAs intake in the diet of older men could reduce resistance exercise‐induced apoptosis (Sheikholeslami‐Vatani et al. 2019).

## 7 Limitations

Despite the growing body of evidence supporting the role of omega‐3 fatty acids and physical exercise in promoting cognitive and physiological health during aging, several limitations are evident across the studies reviewed. Many clinical trials include fewer than 50 participants, limiting statistical power, and the intervention periods typically range from 8 to 12 weeks, which may be insufficient to identify neurobiological changes. Moreover, there is heterogeneity in both supplementation and exercise protocols across studies. Omega‐3 doses vary significantly, as do the types of supplementation. Exercise regimens differed in type, frequency, and intensity, making direct comparisons across studies difficult. We suggest that studies assessing the impact of FAs and exercise on the mental status of elderly people take into consideration the baseline nutritional status of the participants. Such studies must include a placebo control group, and cognition must be assessed with objective assessments. Concerning animal studies, FA doses must be equivalent to those of humans.

## 8 Conclusions

Omega‐3 fatty acids and physical exercise independently influence molecular pathways central to healthy aging, including neuroplasticity, oxidative stress, inflammation, and metabolism. ALA, DHA, and EPA support neuronal membrane integrity, modulate neurotransmitter systems, and attenuate pro‐inflammatory cascades. Exercise enhances BDNF expression, mTOR signaling, hippocampal neurogenesis, and cerebral blood flow. Combined interventions may produce additive or synergistic effects via shared pathways such as BDNF/TrkB, PI3K/Akt, AMPK/SIRT1, and PPAR‐γ. Reported benefits include improved antioxidant defenses, reduced oxidative damage, and moderated neuroinflammation. However, human trial results are inconsistent, with some showing no additional cognitive or anti‐inflammatory advantages from omega‐3 supplementation. Outcomes appear to depend on dosage, nutrient form, exercise modality, sex, age, comorbidities, and genetic factors. Overall, evidence indicates potential but variable synergy in supporting brain and systemic health during aging.

## Author Contributions

**Yikuang Hao:** conceptualization (equal), data curation (equal), investigation (equal), methodology (equal), supervision (equal), validation (equal), visualization (equal), writing – original draft (equal), writing – review and editing (equal). **Sima‐sadat Sabihi:** conceptualization (equal), data curation (equal), investigation (equal), methodology (equal), supervision (equal), validation (equal), visualization (equal), writing – original draft (equal), writing – review and editing (equal).

## Ethics Statement

The authors have nothing to report.

## Consent

The authors have nothing to report.

## Conflicts of Interest

The authors declare no conflicts of interest.

## Data Availability

Data sharing not applicable to this article as no datasets were generated or analyzed during the current study.

## References

[fsn370866-bib-0001] Adolphe, J. L. , S. J. Whiting , B. H. Juurlink , L. U. Thorpe , and J. Alcorn . 2010. “Health Effects With Consumption of the Flax Lignan Secoisolariciresinol Diglucoside.” British Journal of Nutrition 103: 929–938. 20003621 10.1017/S0007114509992753

[fsn370866-bib-0002] Alkadhi, K. A. 2019. “Cellular and Molecular Differences Between Area CA1 and the Dentate Gyrus of the Hippocampus.” Molecular Neurobiology 56: 6566–6580. 30874972 10.1007/s12035-019-1541-2

[fsn370866-bib-0003] Arabzadeh, E. , N. Karimi Nazar , M. Gholami , M. S. Roshani Koosha , and M. Zargani . 2024. “The Effect of Eight Weeks Combined Training With Omega‐3 Supplementation on the Levels of Intercellular Adhesion Molecule‐1 and Vascular Cell Adhesion Molecule‐1 in Older Women.” Clinical Nutrition ESPEN 61: 151–157. 38777428 10.1016/j.clnesp.2024.03.018

[fsn370866-bib-0004] Banich, M. T. , K. L. Mackiewicz , B. E. Depue , A. J. Whitmer , G. A. Miller , and W. Heller . 2009. “Cognitive Control Mechanisms, Emotion and Memory: A Neural Perspective With Implications for Psychopathology.” Neuroscience and Biobehavioral Reviews 33: 613–630. 18948135 10.1016/j.neubiorev.2008.09.010 PMC2865433

[fsn370866-bib-0005] Bhardwaj, K. , N. Verma , R. Trivedi , and S. Bhardwaj . 2015. “Flaxseed Oil and Diabetes: A Systemic Review.” Journal of Medical Sciences 15: 135–138.

[fsn370866-bib-0006] Bischoff‐Ferrari, H. A. , S. Gängler , M. Wieczorek , et al. 2025. “Individual and Additive Effects of Vitamin D, Omega‐3 and Exercise on DNA Methylation Clocks of Biological Aging in Older Adults From the DO‐HEALTH Trial.” Nature Aging 5: 376–385. 39900648 10.1038/s43587-024-00793-y PMC11922767

[fsn370866-bib-0007] Bischoff‐Ferrari, H. A. , B. Vellas , R. Rizzoli , et al. 2020. “Effect of Vitamin D Supplementation, Omega‐3 Fatty Acid Supplementation, or a Strength‐Training Exercise Program on Clinical Outcomes in Older Adults: The DO‐HEALTH Randomized Clinical Trial.” JAMA 324: 1855–1868. 33170239 10.1001/jama.2020.16909 PMC7656284

[fsn370866-bib-0008] Bousquet, M. , M. Saint‐Pierre , C. Julien , N. Salem Jr. , F. Cicchetti , and F. Calon . 2008. “Beneficial Effects of Dietary Omega‐3 Polyunsaturated Fatty Acid on Toxin‐Induced Neuronal Degeneration in an Animal Model of Parkinson's Disease.” FASEB Journal 22: 1213–1225. 18032633 10.1096/fj.07-9677com

[fsn370866-bib-0009] Bruel‐Jungerman, E. , C. Rampon , and S. Laroche . 2007. “Adult Hippocampal Neurogenesis, Synaptic Plasticity and Memory: Facts and Hypotheses.” Reviews in the Neurosciences 18: 93–114. 17593874 10.1515/revneuro.2007.18.2.93

[fsn370866-bib-0010] Cabo, J. , R. Alonso , and P. Mata . 2012. “Omega‐3 Fatty Acids and Blood Pressure.” British Journal of Nutrition 107, no. Suppl 2: S195–S200. 22591893 10.1017/S0007114512001584

[fsn370866-bib-0011] Calder, P. 2001. “N‐3 Polyunsaturated Fatty Acids, Inflammation and Immunity: Pouring Oil on Troubled Waters or Another Fishy Tale?” Nutrition Research 21: 309–341.

[fsn370866-bib-0012] Calder, P. , and R. Grimble . 2002. “Polyunsaturated Fatty Acids, Inflammation and Immunity.” European Journal of Clinical Nutrition 56: S14–S19. 12142955 10.1038/sj.ejcn.1601478

[fsn370866-bib-0013] Cassilhas, R. C. , V. A. Viana , V. Grassmann , et al. 2007. “The Impact of Resistance Exercise on the Cognitive Function of the Elderly.” Medicine and Science in Sports and Exercise 39: 1401–1407. 17762374 10.1249/mss.0b013e318060111f

[fsn370866-bib-0014] Coelho, F. G. , S. Gobbi , C. A. Andreatto , D. I. Corazza , R. V. Pedroso , and R. F. Santos‐Galduróz . 2013. “Physical Exercise Modulates Peripheral Levels of Brain‐Derived Neurotrophic Factor (BDNF): A Systematic Review of Experimental Studies in the Elderly.” Archives of Gerontology and Geriatrics 56: 10–15. 22749404 10.1016/j.archger.2012.06.003

[fsn370866-bib-0015] Coelho‐Júnior, H. J. , A. Álvarez‐Bustos , A. Picca , et al. 2024. “Dietary Intake of Polyunsaturated Fatty Acids Is Associated With Blood Glucose and Diabetes in Community‐Dwelling Older Adults.” Nutrients 16: 4087. 39683480 10.3390/nu16234087 PMC11643427

[fsn370866-bib-0016] Cornish, S. M. , S. B. Myrie , E. M. Bugera , J. E. Chase , D. Turczyn , and M. Pinder . 2018. “Omega‐3 Supplementation With Resistance Training Does Not Improve Body Composition or Lower Biomarkers of Inflammation More So Than Resistance Training Alone in Older Men.” Nutrition Research 60: 87–95. 30527263 10.1016/j.nutres.2018.09.005

[fsn370866-bib-0017] Cotman, C. W. , N. C. Berchtold , and L. A. Christie . 2007. “Exercise Builds Brain Health: Key Roles of Growth Factor Cascades and Inflammation.” Trends in Neurosciences 30: 464–472. 17765329 10.1016/j.tins.2007.06.011

[fsn370866-bib-0018] Csiszar, A. , M. Wang , E. G. Lakatta , and Z. Ungvari . 2008. “Inflammation and Endothelial Dysfunction During Aging: Role of NF‐kappaB.” Journal of Applied Physiology (1985) 105: 1333–1341. 10.1152/japplphysiol.90470.2008 PMC2576023 18599677

[fsn370866-bib-0019] Daďová, K. , M. Petr , J. J. Tufano , et al. 2022. “Calanus Oil Supplementation Does Not Further Improve Short‐Term Memory or Brain‐Derived Neurotrophic Factor in Older Women Who Underwent Exercise Training.” Clinical Interventions in Aging 17: 1227–1236. 35990804 10.2147/CIA.S368079 PMC9384871

[fsn370866-bib-0020] Dalle, S. , E. Van Roie , C. Hiroux , et al. 2021. “Omega‐3 Supplementation Improves Isometric Strength but Not Muscle Anabolic and Catabolic Signaling in Response to Resistance Exercise in Healthy Older Adults.” Journals of Gerontology: Series A 76: 406–414. 10.1093/gerona/glaa309 PMC7907485 33284965

[fsn370866-bib-0021] Dawczynski, C. , K. A. Massey , C. Ness , et al. 2013. “Randomized Placebo‐Controlled Intervention With n‐3 LC‐PUFA‐Supplemented Yoghurt: Effects on Circulating Eicosanoids and Cardiovascular Risk Factors.” Clinical Nutrition 32: 686–696. 23332800 10.1016/j.clnu.2012.12.010

[fsn370866-bib-0022] Dyall, S. C. 2015. “Long‐Chain Omega‐3 Fatty Acids and the Brain: A Review of the Independent and Shared Effects of EPA, DPA and DHA.” Frontiers in Aging Neuroscience 7: 52. 25954194 10.3389/fnagi.2015.00052 PMC4404917

[fsn370866-bib-0023] Eggenberger, P. , M. Wolf , M. Schumann , and E. D. de Bruin . 2016. “Exergame and Balance Training Modulate Prefrontal Brain Activity During Walking and Enhance Executive Function in Older Adults.” Frontiers in Aging Neuroscience 8: 66. 27148041 10.3389/fnagi.2016.00066 PMC4828439

[fsn370866-bib-0024] Erickson, K. I. , R. S. Prakash , M. W. Voss , et al. 2009. “Aerobic Fitness Is Associated With Hippocampal Volume in Elderly Humans.” Hippocampus 19: 1030–1039. 19123237 10.1002/hipo.20547 PMC3072565

[fsn370866-bib-0025] Farooqui, A. A. , and A. A. Farooqui . 2012. “Beneficial Effects of Flaxseed Oil (n‐3 Fatty Acids) on Neurological Disorders.” In Phytochemicals, Signal Transduction, and Neurological Disorders, 57–81. Springer New York.

[fsn370866-bib-0026] Fekete, M. , D. Major , A. Feher , V. Fazekas‐Pongor , and A. Lehoczki . 2024. “Geroscience and Pathology: A New Frontier in Understanding Age‐Related Diseases.” Pathology, Oncology Research: POR 30: 1611623. 38463143 10.3389/pore.2024.1611623 PMC10922957

[fsn370866-bib-0027] Firuzyar, F. , S. Shamlou Kazemi , and A. Hemati Afif . 2023. “The Effect of 8 Weeks of Resistance Training and Consumption of Flaxseed Oil on Some Antioxidant Factors (Catalase and Superoxide Dismutase) in Women With Type 2 Diabetes: A Randomized Controlled Trial.” Journal of Mazandaran University of Medical Sciences 33: 49–60.

[fsn370866-bib-0028] Gajdošová, L. , B. Katrenčíková , V. Borbélyová , and J. Muchová . 2024. “The Effect of Omega‐3 Fatty Acid Supplementation and Exercise on Locomotor Activity, Exploratory Activity, and Anxiety‐Like Behavior in Adult and Aged Rats.” Physiological Research 73: 461–480. 39012176 10.33549/physiolres.935245 PMC11299774

[fsn370866-bib-0029] Giada, M. L. 2010. “Food Applications for Flaxseed and Its Components: Products and Processing.” Recent Patents on Food, Nutrition & Agriculture 2: 181–186. 20858193

[fsn370866-bib-0030] Golanski, J. , P. Szymanska , and M. Rozalski . 2021. “Effects of Omega‐3 Polyunsaturated Fatty Acids and Their Metabolites on Haemostasis—Current Perspectives in Cardiovascular Disease.” International Journal of Molecular Sciences 22: 2394. 33673634 10.3390/ijms22052394 PMC7957531

[fsn370866-bib-0031] Goyens, P. L. , M. E. Spilker , P. L. Zock , M. B. Katan , and R. P. Mensink . 2006. “Conversion of Alpha‐Linolenic Acid in Humans Is Influenced by the Absolute Amounts of Alpha‐Linolenic Acid and Linoleic Acid in the Diet and Not by Their Ratio.” American Journal of Clinical Nutrition 84: 44–53. 16825680 10.1093/ajcn/84.1.44

[fsn370866-bib-0032] Gunning‐Dixon, F. M. , A. M. Brickman , J. C. Cheng , and G. S. Alexopoulos . 2009. “Aging of Cerebral White Matter: A Review of MRI Findings.” International Journal of Geriatric Psychiatry 24: 109–117. 18637641 10.1002/gps.2087 PMC2631089

[fsn370866-bib-0033] Hall, C., 3rd , M. C. Tulbek , and Y. Xu . 2006. “Flaxseed.” Advances in Food and Nutrition Research 51: 1–97. 17011474 10.1016/S1043-4526(06)51001-0

[fsn370866-bib-0034] Hamazaki, J. , and S. Murata . 2024. “Relationships Between Protein Degradation, Cellular Senescence, and Organismal Aging.” Journal of Biochemistry 175: 473–480. 38348509 10.1093/jb/mvae016 PMC11058314

[fsn370866-bib-0035] Harris, W. S. 1997. “N‐3 Fatty Acids and Serum Lipoproteins: Human Studies.” American Journal of Clinical Nutrition 65: 1645s–1654s. 9129504 10.1093/ajcn/65.5.1645S

[fsn370866-bib-0036] Haß, U. , B. Kochlik , C. Herpich , S. Rudloff , and K. Norman . 2022. “Effects of an Omega‐3 Supplemented, High‐Protein Diet in Combination With Vibration and Resistance Exercise on Muscle Power and Inflammation in Old Adults: A Pilot Randomized Controlled Trial.” Nutrients 14: 4274. 36296958 10.3390/nu14204274 PMC9609960

[fsn370866-bib-0037] Hausdorff, J. M. , G. Yogev , S. Springer , E. S. Simon , and N. Giladi . 2005. “Walking Is More Like Catching Than Tapping: Gait in the Elderly as a Complex Cognitive Task.” Experimental Brain Research 164: 541–548. 15864565 10.1007/s00221-005-2280-3

[fsn370866-bib-0038] Hertzog, C. , A. F. Kramer , R. S. Wilson , and U. Lindenberger . 2008. “Enrichment Effects on Adult Cognitive Development: Can the Functional Capacity of Older Adults be Preserved and Enhanced?” Psychological Science in the Public Interest 9: 1–65. 26162004 10.1111/j.1539-6053.2009.01034.x

[fsn370866-bib-0039] Hoirisch‐Clapauch, S. , and A. E. Nardi . 2015. “Improvement of Psychotic Symptoms and the Role of Tissue Plasminogen Activator.” International Journal of Molecular Sciences 16: 27550–27560. 26593907 10.3390/ijms161126053 PMC4661911

[fsn370866-bib-0040] Holtzer, R. , R. Friedman , R. B. Lipton , M. Katz , X. Xue , and J. Verghese . 2007. “The Relationship Between Specific Cognitive Functions and Falls in Aging.” Neuropsychology 21: 540–548. 17784802 10.1037/0894-4105.21.5.540 PMC3476056

[fsn370866-bib-0041] Holtzer, R. , J. Verghese , X. Xue , and R. B. Lipton . 2006. “Cognitive Processes Related to Gait Velocity: Results From the Einstein Aging Study.” Neuropsychology 20: 215–223. 16594782 10.1037/0894-4105.20.2.215

[fsn370866-bib-0042] Hussein, N. , E. Ah‐Sing , P. Wilkinson , C. Leach , B. A. Griffin , and D. J. Millward . 2005. “Long‐Chain Conversion of [13C]Linoleic Acid and Alpha‐Linolenic Acid in Response to Marked Changes in Their Dietary Intake in Men.” Journal of Lipid Research 46: 269–280. 15576848 10.1194/jlr.M400225-JLR200

[fsn370866-bib-0043] Ikegaya, Y. , Y. Ishizaka , and N. Matsuki . 2002. “BDNF Attenuates Hippocampal LTD via Activation of Phospholipase C: Implications for a Vertical Shift in the Frequency–Response Curve of Synaptic Plasticity.” European Journal of Neuroscience 16: 145–148. 12153539 10.1046/j.1460-9568.2002.02051.x

[fsn370866-bib-0044] Jaca, A. , S. Durão , and J. Harbron . 2020. “Omega‐3 Fatty Acids for the Primary and Secondary Prevention of Cardiovascular Disease.” South African Medical Journal 110: 1158–1159. 33403957 10.7196/SAMJ.2020.v110i12.14730

[fsn370866-bib-0045] Jahanshiri, N. , and N. Bijeh . 2020. “Effect of 8‐Week Combined Training (Endurance, Strength) With Flaxseed Consumption on Insulin Resistance and Blood Pressure in Postmenopausal Women.” Journal of Sabzevar University of Medical Sciences 27: 173–181.

[fsn370866-bib-0046] Jang, W. Y. , M. Y. Kim , and J. Y. Cho . 2022. “Antioxidant, Anti‐Inflammatory, Anti‐Menopausal, and Anti‐Cancer Effects of Lignans and Their Metabolites.” International Journal of Molecular Sciences 23: 15482. 36555124 10.3390/ijms232415482 PMC9778916

[fsn370866-bib-0047] Janssen, C. I. , and A. J. Kiliaan . 2014. “Long‐Chain Polyunsaturated Fatty Acids (LCPUFA) From Genesis to Senescence: The Influence of LCPUFA on Neural Development, Aging, and Neurodegeneration.” Progress in Lipid Research 53: 1–17. 24334113 10.1016/j.plipres.2013.10.002

[fsn370866-bib-0048] Kennedy, K. M. , and N. Raz . 2009. “Aging White Matter and Cognition: Differential Effects of Regional Variations in Diffusion Properties on Memory, Executive Functions, and Speed.” Neuropsychologia 47: 916–927. 19166865 10.1016/j.neuropsychologia.2009.01.001 PMC2643310

[fsn370866-bib-0049] Kleigrewe, K. , M. Haack , M. Baudin , et al. 2022. “Dietary Modulation of the Human Gut Microbiota and Metabolome With Flaxseed Preparations.” International Journal of Molecular Sciences 23: 10473. 36142393 10.3390/ijms231810473 PMC9499670

[fsn370866-bib-0050] Knaepen, K. , M. Goekint , E. M. Heyman , and R. Meeusen . 2010. “Neuroplasticity ‐ Exercise‐Induced Response of Peripheral Brain‐Derived Neurotrophic Factor: A Systematic Review of Experimental Studies in Human Subjects.” Sports Medicine 40: 765–801. 20726622 10.2165/11534530-000000000-00000

[fsn370866-bib-0051] Kokoli, A. , S. Lavrentiadou , I. Zervos , et al. 2017. “Dietary Omega‐3 Polyunsaturated Fatty Acids Induce Plasminogen Activator Activity and DNA Damage in Rabbit Spermatozoa.” Andrologia 49: e12776. 10.1111/and.12776 28217940

[fsn370866-bib-0052] Kris‐Etherton, P. M. , W. S. Harris , and L. J. Appel . 2003. “Fish Consumption, Fish Oil, Omega‐3 Fatty Acids, and Cardiovascular Disease.” Arteriosclerosis, Thrombosis, and Vascular Biology 23: e20–e30. 12588785 10.1161/01.atv.0000038493.65177.94

[fsn370866-bib-0053] Kumar, M. S. , and S. Devaraja . 2022. “Flaxseed Has a Pronounced Effect on Gut Microbiota.” In Microbiome, Immunity, Digestive Health and Nutrition, 417–430. Elsevier.

[fsn370866-bib-0054] Leuner, B. , and E. Gould . 2010. “Structural Plasticity and Hippocampal Function.” Annual Review of Psychology 61: 111–140. 10.1146/annurev.psych.093008.100359 PMC3012424 19575621

[fsn370866-bib-0055] Logan, S. L. , and L. L. Spriet . 2015. “Omega‐3 Fatty Acid Supplementation for 12 Weeks Increases Resting and Exercise Metabolic Rate in Healthy Community‐Dwelling Older Females.” PLoS One 10: e0144828. 26679702 10.1371/journal.pone.0144828 PMC4682991

[fsn370866-bib-0056] Lopez‐Franco, A. , A. Y. Alanis , C. Lopez‐Franco , N. Arana‐Daniel , and M. Lopez‐Franco . 2018. “Emotional System in Complex Cognitive Activities of Working Memory: A Literature Review of Its Role.” Journal of Integrative Neuroscience 17: 679–693. 30103346 10.3233/JIN-180095

[fsn370866-bib-0057] Mattson, M. P. 2004. “Pathways Towards and Away From Alzheimer's Disease.” Nature 430: 631–639. 15295589 10.1038/nature02621 PMC3091392

[fsn370866-bib-0058] Melchor, J. P. , R. Pawlak , and S. Strickland . 2003. “The Tissue Plasminogen Activator‐Plasminogen Proteolytic Cascade Accelerates Amyloid‐β (Aβ) Degradation and Inhibits Aβ‐Induced Neurodegeneration.” Journal of Neuroscience 23: 8867–8871. 14523088 10.1523/JNEUROSCI.23-26-08867.2003 PMC6740393

[fsn370866-bib-0059] Mengelberg, A. , J. Leathem , J. Podd , S. Hill , and C. Conlon . 2022. “The Effects of Docosahexaenoic Acid Supplementation on Cognition and Well‐Being in Mild Cognitive Impairment: A 12‐Month Randomised Controlled Trial.” International Journal of Geriatric Psychiatry 37. 10.1002/gps.5707. PMC9321856 35373862

[fsn370866-bib-0060] Mizui, T. , Y. Ishikawa , H. Kumanogoh , et al. 2015. “BDNF Pro‐Peptide Actions Facilitate Hippocampal LTD and Are Altered by the Common BDNF Polymorphism Val66Met.” Proceedings of the National Academy of Sciences of the United States of America 112: E3067–E3074. 26015580 10.1073/pnas.1422336112 PMC4466729

[fsn370866-bib-0061] Mueed, A. , P. Madjirebaye , S. Shibli , and Z. Deng . 2022. “Flaxseed Peptides and Cyclolinopeptides: A Critical Review on Proteomic Approaches, Biological Activity, and Future Perspectives.” Journal of Agricultural and Food Chemistry 70: 14600–14612. 36355404 10.1021/acs.jafc.2c06769

[fsn370866-bib-0062] Mueed, A. , S. Shibli , S. A. Korma , P. Madjirebaye , T. Esatbeyoglu , and Z. Deng . 2022. “Flaxseed Bioactive Compounds: Chemical Composition, Functional Properties, Food Applications and Health Benefits‐Related Gut Microbes.” Foods (Basel, Switzerland) 11: 3307. 37431051 10.3390/foods11203307 PMC9602266

[fsn370866-bib-0063] Osmakov, D. I. , A. P. Kalinovskii , O. A. Belozerova , Y. A. Andreev , and S. A. Kozlov . 2022. “Lignans as Pharmacological Agents in Disorders Related to Oxidative Stress and Inflammation: Chemical Synthesis Approaches and Biological Activities.” International Journal of Molecular Sciences 23: 6031. 35682715 10.3390/ijms23116031 PMC9181380

[fsn370866-bib-0064] Paduchová, Z. , L. Gajdošová , B. Katrenčíková , et al. 2024. “Synergistic Effects of Omega‐3 Fatty Acids and Physical Activity on Oxidative Stress Markers and Antioxidant Mechanisms in Aged Rats.” Nutrients 17: 96. 39796529 10.3390/nu17010096 PMC11723026

[fsn370866-bib-0065] Pang, P. T. , H. K. Teng , E. Zaitsev , et al. 2004. “Cleavage of proBDNF by tPA/Plasmin Is Essential for Long‐Term Hippocampal Plasticity.” Science 306: 487–491. 15486301 10.1126/science.1100135

[fsn370866-bib-0066] Pelegrino, A. F. , M. Attarha , P. J. Toussaint , et al. 2025. “Cholinergic Neurotransmission in the Anterior Cingulate Cortex Is Associated With Cognitive Performance in Healthy Older Adults: Baseline Characteristics of the Improving Neurological Health in Aging via Neuroplasticity‐Based Computerized Exercise (INHANCE) Trial.” NeuroImage Reports 5: 100234. 40191405 10.1016/j.ynirp.2025.100234 PMC11970925

[fsn370866-bib-0067] Peterson, J. , J. Dwyer , H. Adlercreutz , A. Scalbert , P. Jacques , and M. L. McCullough . 2010. “Dietary Lignans: Physiology and Potential for Cardiovascular Disease Risk Reduction.” Nutrition Reviews 68: 571–603. 20883417 10.1111/j.1753-4887.2010.00319.x PMC2951311

[fsn370866-bib-0068] Phillips, C. 2017. “Lifestyle Modulators of Neuroplasticity: How Physical Activity, Mental Engagement, and Diet Promote Cognitive Health During Aging.” Neural Plasticity 2017: 3589271. 28695017 10.1155/2017/3589271 PMC5485368

[fsn370866-bib-0069] Rahman, S. 2020. “Mitochondrial Disease in Children.” Journal of Internal Medicine 287: 609–633. 32176382 10.1111/joim.13054

[fsn370866-bib-0070] Schättin, A. , R. Arner , F. Gennaro , and E. D. de Bruin . 2016. “Adaptations of Prefrontal Brain Activity, Executive Functions, and Gait in Healthy Elderly Following Exergame and Balance Training: A Randomized‐Controlled Study.” Frontiers in Aging Neuroscience 8: 278. 27932975 10.3389/fnagi.2016.00278 PMC5120107

[fsn370866-bib-0071] Schättin, A. , C. Baier , D. Mai , V. Klamroth‐Marganska , I. Herter‐Aeberli , and E. D. de Bruin . 2019. “Effects of Exergame Training Combined With Omega‐3 Fatty Acids on the Elderly Brain: A Randomized Double‐Blind Placebo‐Controlled Trial.” BMC Geriatrics 19: 1–16. 30866834 10.1186/s12877-019-1084-4 PMC6416848

[fsn370866-bib-0072] Scherder, E. , L. Eggermont , C. Visscher , P. Scheltens , and D. Swaab . 2011. “Understanding Higher Level Gait Disturbances in Mild Dementia in Order to Improve Rehabilitation: ‘last In‐First Out’.” Neuroscience and Biobehavioral Reviews 35: 699–714. 20833200 10.1016/j.neubiorev.2010.08.009

[fsn370866-bib-0073] Schliebs, R. , and T. Arendt . 2011. “The Cholinergic System in Aging and Neuronal Degeneration.” Behavioural Brain Research 221: 555–563. 21145918 10.1016/j.bbr.2010.11.058

[fsn370866-bib-0074] Sebastian Monasor, L. , S. A. Müller , A. V. Colombo , et al. 2020. “Fibrillar Aβ Triggers Microglial Proteome Alterations and Dysfunction in Alzheimer Mouse Models.” eLife 9: 9. 10.7554/eLife.54083 PMC7279888 32510331

[fsn370866-bib-0075] Sheikholeslami‐Vatani, D. , S. Ahmadi , and H. Faraji . 2019. “The Effects of Omega‐3 and Branched‐Chain Amino Acids Supplementation on Serum Apoptosis Markers Following Acute Resistance Exercise in Old Men.” Journal of Aging and Physical Activity 27: 198–204. 30117365 10.1123/japa.2017-0404

[fsn370866-bib-0076] Sheridan, P. L. , J. Solomont , N. Kowall , and J. M. Hausdorff . 2003. “Influence of Executive Function on Locomotor Function: Divided Attention Increases Gait Variability in Alzheimer's Disease.” Journal of the American Geriatrics Society 51: 1633–1637. 14687395 10.1046/j.1532-5415.2003.51516.x

[fsn370866-bib-0077] Shibabaw, T. 2021. “Omega‐3 Polyunsaturated Fatty Acids: Anti‐Inflammatory and Anti‐Hypertriglyceridemia Mechanisms in Cardiovascular Disease.” Molecular and Cellular Biochemistry 476: 993–1003. 33179122 10.1007/s11010-020-03965-7

[fsn370866-bib-0078] Sidhu, K. S. 2003. “Health Benefits and Potential Risks Related to Consumption of Fish or Fish Oil.” Regulatory Toxicology and Pharmacology 38: 336–344. 14623484 10.1016/j.yrtph.2003.07.002

[fsn370866-bib-0079] Singh, K. K. , D. Mridula , J. Rehal , and P. Barnwal . 2011. “Flaxseed: A Potential Source of Food, Feed and Fiber.” Critical Reviews in Food Science and Nutrition 51: 210–222. 21390942 10.1080/10408390903537241

[fsn370866-bib-0080] Sobhani, V. , R. Shakibaei , M. Gholami , and E. Arabzadeh . 2024. “Effect of Eight Weeks of Combined Exercise and Omega‐3 Supplementation on the Levels of Inflammatory and Anti‐Inflammatory Cytokines in Elderly Women.” Comparative Exercise Physiology 20: 273–282.

[fsn370866-bib-0081] Stanmore, E. , B. Stubbs , D. Vancampfort , E. D. de Bruin , and J. Firth . 2017. “The Effect of Active Video Games on Cognitive Functioning in Clinical and Non‐Clinical Populations: A Meta‐Analysis of Randomized Controlled Trials.” Neuroscience and Biobehavioral Reviews 78: 34–43. 28442405 10.1016/j.neubiorev.2017.04.011

[fsn370866-bib-0082] Ungvari, Z. , S. Tarantini , A. J. Donato , V. Galvan , and A. Csiszar . 2018. “Mechanisms of Vascular Aging.” Circulation Research 123: 849–867. 30355080 10.1161/CIRCRESAHA.118.311378 PMC6248882

[fsn370866-bib-0083] Venezia, A. C. , E. Quinlan , and S. M. Roth . 2017. “A Single Bout of Exercise Increases Hippocampal Bdnf: Influence of Chronic Exercise and Noradrenaline.” Genes, Brain, and Behavior 16: 800–811. 28556463 10.1111/gbb.12394 PMC5677569

[fsn370866-bib-0084] Voss, M. W. , C. Vivar , A. F. Kramer , and H. van Praag . 2013. “Bridging Animal and Human Models of Exercise‐Induced Brain Plasticity.” Trends in Cognitive Sciences 17: 525–544. 24029446 10.1016/j.tics.2013.08.001 PMC4565723

[fsn370866-bib-0085] Wu, C. K. , L. Thal , D. Pizzo , L. Hansen , E. Masliah , and C. Geula . 2005. “Apoptotic Signals Within the Basal Forebrain Cholinergic Neurons in Alzheimer's Disease.” Experimental Neurology 195: 484–496. 16085017 10.1016/j.expneurol.2005.06.020

[fsn370866-bib-0086] Yabluchanskiy, A. , Z. Ungvari , A. Csiszar , and S. Tarantini . 2018. “Advances and Challenges in Geroscience Research: An Update.” Physiology International 105: 298–308. 30587027 10.1556/2060.105.2018.4.32 PMC9341286

[fsn370866-bib-0087] Yang, C. E. , T. M. Yeh , C. D. Chang , and W. L. Shih . 2021. “Chinese Soft‐Shelled Turtle Oil in Combination With Swimming Training Improves Spatial Memory and Sports Performance of Aging Rats.” Frontiers in Physiology 12: 660552. 34122132 10.3389/fphys.2021.660552 PMC8194302

[fsn370866-bib-0088] Yang, J. , N. Sáinz , E. Félix‐Soriano , et al. 2021. “Effects of Long‐Term DHA Supplementation and Physical Exercise on Non‐Alcoholic Fatty Liver Development in Obese Aged Female Mice.” Nutrients 13: 501. 33546405 10.3390/nu13020501 PMC7913512

[fsn370866-bib-0089] Yang, J. , C. Wen , Y. Duan , et al. 2021. “The Composition, Extraction, Analysis, Bioactivities, Bioavailability and Applications in Food System of Flaxseed ( *Linum usitatissimum* L.) Oil: A Review.” Trends in Food Science & Technology 118: 252–260.

[fsn370866-bib-0090] Yavari, M. , N. S. Kalupahana , B. N. Harris , et al. 2025. “Mechanisms Linking Obesity, Insulin Resistance, and Alzheimer's Disease: Effects of Polyphenols and Omega‐3 Polyunsaturated Fatty Acids.” Nutrients 17: 1203. 40218960 10.3390/nu17071203 PMC11990358

